# Insights into the Procoagulant Profile of Patients with Systemic Lupus Erythematosus without Antiphospholipid Antibodies

**DOI:** 10.3390/jcm9103297

**Published:** 2020-10-14

**Authors:** Elena Monzón Manzano, Ihosvany Fernández-Bello, Raúl Justo Sanz, Ángel Robles Marhuenda, Francisco Javier López-Longo, Paula Acuña, María Teresa Álvarez Román, Víctor Jiménez Yuste, Nora V. Butta

**Affiliations:** 1 Hematology Unit, University Hospital La Paz-Idipaz, Paseo de la Castellana 231, 28046 Madrid, Spain; elenamonzonmanzano@hotmail.com (E.M.M.); ihosvanyf@yahoo.es (I.F.-B.); rauljustosanz@gmail.com (R.J.S.); paulaacbu@gmail.com (P.A.); talvarezroman@gmail.com (M.T.Á.R.); vjyuste@gmail.com (V.J.Y.); 2 Internal Medicine Unit, Hospital Universitario La Paz-IdiPAZ, 28046 Madrid, Spain; aroblesmarhuenda@gmail.com; 3 Rheumatology Unit, University Hospital Gregorio Marañón, 28007 Madrid, Spain; hemostasia.hulp@gmail.com; 4 Faculty of Medicine, Universidad Autónoma de Madrid, 28029 Madrid, Spain

**Keywords:** systemic lupus erythematosus, thromboelastometry, thrombin generation, neutrophil extracellular traps

## Abstract

We aimed to identify the key players in the prothrombotic profile of patients with systemic lupus erythematosus (SLE) not mediated by antiphospholipid antibodies, as well as the potential utility of global coagulation tests to characterize hemostasis in these patients. Patients with SLE without antiphospholipid antibodies and without signs of thrombosis were included. The kinetics of clot formation were determined by ROTEM^®^. Platelet activation markers were determined by flow cytometry. Thrombin generation associated with Neutrophil Extracellular Traps (NETs) and microparticles (MPs) was measured by calibrated automated thrombogram (CAT). The plasma levels of PAI-1 were also determined. ROTEM^®^ showed a procoagulant profile in SLE patients. SLE patients had activated platelets and more leukocyte/platelet aggregates at basal conditions. The plasma PAI-1 and platelet aggregates correlated with several ROTEM^®^ parameters. The thrombin generation associated withthe tissue factor (TF) content of MPs and with NETs was increased. Our results suggest the utility of global tests for studying hemostasis in SLE patients because they detect their procoagulant profile, despite having had neither antiphospholipid antibodies nor any previous thrombotic event. A global appraisal of hemostasis should, if possible, be incorporated into clinical practice to detect the risk of a thrombotic event in patients with SLE and to consequently act to prevent its occurrence.

## 1. Introduction

Systemic lupus erythematosus (SLE) is a potentially fatal multiorgan inflammatory immune-mediated disease that primarily affects females. The disease is characterized by the production of antibodies against various tissues, which triggers a wide variety of cutaneous and systemic manifestations that in many cases become serious, compromising the patient’s life. Thrombosis contributes to substantial morbidity and mortality in patients with SLE due to a complex interplay between traditional risk factors and the dysregulation of autoimmunity. Up to 15% of patients with SLE have had myocardial infarction [1], and approximately 20–30% of deaths in patients with SLE are due to cardiovascular disease (CVD) [2,3]. Conflicting data on the mechanisms involved in the increase in risk and in the prediction of CVD complicate prevention of its occurrence.

The duration of the disease correlates with the degree of cardiovascular involvement [4], suggesting that chronic exposure to immune system dysregulation contributes to the development of CDV in these patients. The proposed predictors of cardiovascular events in this population are dyslipidemia, hypertension, a family medical history of coronary artery disease (CAD), and smoking. In addition, although the presence of antiphospholipid and anticardiolipin antibodies and lupus anticoagulant is correlated with the occurrence of cardiovascular events, 40% of SLE thrombosis cases are autoantibody-negative [5,6], suggesting the involvement of other factors.Thus, we aim to identify the key players in the prothrombotic profile of patients with SLE not mediated by antiphospholipid antibodies.

Recently, there has been growing interest in the use of global coagulation tests to evaluate hypercoagulable states [7,8]. Among them, rotational thromboelastometry (ROTEM^®^), a viscoelastometric clotting test that measures the kinetics of clot formation and fibrinolysis, and calibrated automated thrombogram (CAT), a thrombin generation test that quantifies thrombin generation, are the most widely used. Given that hemostasis is the consequence of the relationship between various cells, coagulation factors, and plasma components, we considered that these tests would be a good approach to evaluate the hypercoagulable condition in SLE. Therefore, we investigated the potential utility of ROTEM^®^ and CAT in the characterization of the procoagulant state in SLE not mediated by antiphospholipid antibodies, giving a new insight into the relationship between different factors involved in this pathology.

## 2. Materials and Methods

### 2.1. Participants and Study Design

This study was approved by the ethics committees of two hospitals: Gregorio Marañón University Hospital (Code 324/14) and La Paz University Hospital (Code PI-3293). All the included patients had been diagnosed with SLE according to the American College of Rheumatology (ACR) criteria for SLE [9]. The global disease activity was measured with the Systemic Lupus Erythematosus Activity Index 2000 (SLEDAI-2K).

Exclusion criteria were infection with hepatitis C virus or human immunodeficiency virus; alcohol abuse or addiction; oral contraceptive intake or hormonal therapy (excepting steroids as an immunosuppressive treatment for SLE); patients with antiphospholipid antibodies (lupus anticoagulant, anti-β2-GPI, and anticardiolipin antibodies); a history of acute myocardial infarction, angina, or CAD; diabetes, hyperlipemia, or uncontrolled arterial hypertension; overweight defined by a body mass index ≥ 30 kg/m^2^; smoking in the 12 months before our study; pregnancy in the previous 3 months prior to the study; or cancer.

Patients older than 18 years who fulfilled 4 or more ACR criteria, with a titer of antinuclear antibodies ≥1:80 and/or anti-double-stranded DNA antibody (anti-dsDNA antibody) levels ≥ 30 UI/mL, with a stable standard SLE therapy for the last 30 days, and who signed written informed consent were included in this study.

### 2.2. Collection and Preparation of Samples

Human peripheral blood samples were collected in tubes containing 3.2% trisodium citrate (BD Vacutainer, Madrid, Spain). Platelet-rich plasma (PRP) was prepared within 60 min of blood collection by centrifugation (150 g for 20 min at 23 °C). To obtain platelet-poor plasma (PPP), the PRP was centrifuged twiceat 23 °C, first at 1500 g for 15 min and then at 13,000 g for 2 min.

Acid-citrate-dextrose (1:10) was added to the top two-third volumes of PRP and centrifuged at 650 g for 10 min at 23 °C to obtain washed platelets. The pellet was then resuspended in an equal volume of 4-(2-hydroxyethyl)-1-piperazineethanesulfonic acid (HEPES) buffer (10 mM of HEPES, 145 mM of NaCl, 5 mM of KCl, and 1 mM of MgSO_4_, pH 7.4).

For serum preparation, peripheral blood was collected in serum tubes (BD Vacutainer, Plymouth, UK) and separated by centrifuging clotted blood (2500 g for 15 min at 23 °C).

Plasma and serum aliquots were stored at −80 °C until analysis.

### 2.3. Cell Count and Biochemical Parameters

The blood cell count was performed using a Coulter AcT Diff cell counter (Beckman Coulter, Madrid, Spain). The plasminogen activator inhibitor type 1 (PAI-1) (Invitrogen, Vienna, Austria) levels were determined in serum or plasma according to the manufacturer’s instructions and measured in a Multiskan FC microplate photometer (ThermoScientific, Madrid, Spain).

The cell-free DNA (cfDNA) was determined in PPP by the Quant-iT™ PicoGreen dsDNA assay (Thermo Fisher Scientific, Waltham, MA, USA) according to the manufacturer’s instructions.

C-reactive protein (CRP), serum complement C3 and C4, erythrocyte sedimentation rate (ESR), creatinine, 24-hproteinuria, and anti-DNA titer were only determined in the SLE groups.

### 2.4. Rotational Thromboelastometry

The kinetics of clot formation and fibrinolysis were assessed by rotational thromboelastometry (ROTEM^®^, Pentapharm, Munich, Germany) with the recalcification of whole blood (NATEM^®^test).

The following parameters were recorded: clotting time (CT) (time from the start of clot formation until an amplitude of 2 mm, in seconds); alpha angle (α) (the slope of the tangent line to the clotting curve through the 20 mm amplitude that reflects the rate of fibrin polymerization, in degrees); the clot firmness X min after CT; the maximal clot firmness (MCF, in mm); and clot lysis as the percentage of clot lysedafter 60 min.

### 2.5. Analysis of Platelet Activation and Platelet Receptors

PRP was diluted 1:5 with HEPES buffer and incubated with either buffer, 100 µmol/L of thrombin receptor-activating peptide (TRAP)-6 (Bachem, Switzerland), or 10 µmol/L of adenosine diphosphate (ADP, Sigma, Madrid, Spain) at room temperature (RT). Later, fluorescein-isothiocyanate (FITC)-PAC1 (BD, Madrid, Spain), a monoclonal antibody (mAb) that recognizes only the activated conformation of fibrinogen receptor, FITC-anti P-selectin mAb (BD Pharmingen, San Diego, CA, USA), or FITC anti-CD63 mAb (BD, Madrid, Spain) were added and incubated for 15 min at RT. To determine the platelet receptors, diluted PRP was incubated with phycoerythrin (PE) anti-CD41 mAb (Biocytex, Marseille, France) or FITC anti-CD61mAb (BD, Madrid, Spain)—which recognized, respectively, the αIIb and β3 subunits of fibrinogen receptor—or it was incubated with FITC anti-CD42a mAb (BD, Madrid, Spain) or anti-CD42b mAb (BD Pharmingen, San Diego, CA, USA), against, respectively, the GPIX and GPIbα subunits of von Willebrand factor (vWF) receptor. These samples were analyzed using a FACScan flow cytometer (BD Biosciences, Madrid, Spain) after being diluted in 1:6 HEPES buffer.

### 2.6. Determination of Platelet-Leukocyte Aggregates

To determine the platelet-leukocyte aggregates, whole blood was diluted 1:10 in HEPES buffer and coincubated with FITC anti-CD45 mAb (BD Pharmingen, San Diego, CA, USA), PE anti-CD41 mAb, and 50 µM of TRAP or 40 µM of ADP for 15 min at RT in the dark. Platelet-leukocyte aggregates were defined as leukocytes positive for CD41.

### 2.7. Determination of Phosphatidylserine Exposure on Platelet Surface and Caspase Activity

The surface exposure of phosphatidylserine (PS) in washed platelets was assessed by measuring the binding of (FITC)-labeled Annexin V (BD Pharmingen, San Diego, CA, USA). Washed platelets were resuspended in annexinV binding buffer (10 mM of HEPES, 10 mM of NaOH, 140 mM of NaCl, 2.5 mM of CaCl_2_, pH 7.4) and labeled with FITC-annexinV. After incubation for 15 min at RT in the dark, the samples were analyzed by flow cytometry.

To analyze the caspase-3, -7, -8, and -9 activity, PRP was diluted 10-fold with isotonic HEPES buffer containing 2 mM of CaCl_2_ and 2 mM of Gly-Pro-Arg-Pro acetate (Sigma Aldrich, Madrid, Spain) to prevent fibrin formation, and either FAM-DEVD-FMK, FAM-LETD-FMK, or FAM-LEHD-FMK (Millipore, Madrid, Spain). The samples were analyzed by flow cytometry.

### 2.8. Calibrated Automated Thrombogram

The procoagulant activity of MPs associated with their content of either tissue factor (TF) or PS was determined, respectively, with MP reagent (4 µM of phospholipids) or PRP reagent (1 pM of recombinant human TF) by calibrated automated thrombogram (CAT). All CAT reagents were from Diagnostica Stago (Madrid, Spain). The thrombin generation was determined with a Fluoroskan FL instrument (ThermoLabsystems, Helsinki, Finland) under the control of Thrombinoscope software, version 3.6 (Thrombinoscope BV, Maastricht, Holand), filtered for excitation at 390 nm and emission at 460 nm.

The following parameters were determined: lagtime (LT) (time from the start of the assay until 10 nM of thrombin is formed, in min), time to peak (ttPeak) (time required to reach the maximum thrombin concentration, in min), peak height (Peak) (maximum thrombin concentration reached, in nM), and endogenous thrombin potential (ETP) (the total amount of thrombin generated over time, in nMxmin).

### 2.9. Neutrophil Isolation and Generation of Neutrophil Extracellular Traps

Neutrophils were isolated from 10 mL of whole blood from controls and from patients with SLE using a Percoll gradient centrifuged at 500 g for 25 min at 5 °C. The isolated neutrophils (2.5 × 10^6^ cells/mL) were incubated with and without 100 nM of phorbol 12-myristate 13-acetate (PMA) (Sigma-Aldrich, Madrid, Spain) for 45 min at 37 °C in Roswell Park Memorial Institute (RPMI)-1640 medium (Invitrogen, Madrid, Spain). Later, the samples were centrifuged at 5000 g for 3 min and resuspended in PRP from healthy controls. The Neutrophil Extracellular Traps (NETs) formation was verified by fluorescence microscopy.

### 2.10. Assessment of Neutrophil Extracellular Trap Generation by Fluorescence Microscopy

Neutrophils were seeded on 12 mm cover glasses pretreated with poly-L-lysine (Sigma-Aldrich, Sweden) in 24-well plates in 500 µL of RPMI-1640 medium with and without 100 nM of PMA, for 45 min at 37 °C. The samples were fixed with a final concentration of 2% paraformaldehyde for 15 min at RT. Then, the preparations were blocked, adding 2% bovine serum albumin–phosphate-buffered saline for 45 min at RT and incubated first with a 1:300 dilution of rabbit anti-human myeloperoxidase (Dako, Madrid, Spain) and then with Alexa Fluor 488 goat anti-rabbit IgG (Invitrogen, Madrid, Spain) for 45 min at RT in dark. Finally, the samples were embedded in mounting medium with 4′,6-diamidino-2-phenylindole (Vector Laboratories, Burlingame, CA, USA) and kept at 4 °C in the dark until visualization with fluorescence microscopy using the Nikon Eclipse 90i microscope.

### 2.11. Thrombin Generation Associated with Neutrophil Extracellular Traps

Blood from healthy controls was drawn in 2 tubes with citrate as an anticoagulant (Vacutainer, Madrid, Spain) and in 2 tubes with citrate plus 50 µg/µL of corn trypsin inhibitor (CTI) (Cell Systems Biotechnologie, Troisdorf, Germany) to inhibit activated factor XII (FXII). One of each kind of tube was centrifuged to obtain PRP, and the others were centrifuged for PPP. PRP either with or without CTI was adjusted to 1 × 10^5^ platelets/µL with the corresponding PPP. Neutrophils were isolated from healthy controls and from patients with SLE from citrated blood, as described above. Neutrophils were added to wells at a final concentration of 2.5 × 10^5^ cells to 40 μL aliquots of PRP from healthy controls, with and without CTI, to perform CAT experiments after incubation for 30 min at 37 °C with either buffer or 100 nM of PMA without the addition of any trigger.

### 2.12. Statistical Analysis

The Shapiro–Wilk test was used to assess the distribution of the data, and the results were expressed as mean ± SD or median (p25–p75) depending on the distribution. The differences between the 2 groups were assessed using the 2-tailed unpaired Student’s t-test or the non-parametric Mann–Whitney U-test, as appropriate. The correlation analysis was performed using Pearson’s or Spearman’s test. The GraphPad Prism 5 software (GraphPad Software version 5.03, GraphPad Software, San Diego, CA, USA) was used for all the statistical analyses, and significance was set at *p ≤* 0.05.

## 3. Results

### 3.1. Experimental Results

#### 3.1.1. Features of the Patients with SLE

The study was performed on 32 patients with SLE treated at the Rheumatology Unit of the Gregorio Marañón University Hospital and at the Internal Medicine Unit of La Paz University Hospital, with a median age of 41.9 ± 12.9 years, who were recruited after signing informed consent. Eighty-eight sex- and age-matched healthy controls, with a mean age of 38.3 ± 11.6 years, were recruited as controls at the Blood Donation Center of La Paz University Hospital. The study was performed between January 2017 and October 2019. None of the patients had a history or signs or symptoms of thrombosis at inclusion.

A summary of the clinical and demographic data of the patients with SLE is shown in Table 1.

Lymphocytes, erythrocytes, granulocytes, leukocytes, and platelet counts were reduced in the patients with SLE (Table 2). The CRP, C3 and C4 levels, ESR, creatinine, proteinuria (24-h), anti-DNA, IgA, IgM, and IgG levels were determined in most of the patients (Table 2).

#### 3.1.2. Global Hemostasis in Patients with Systemic Lupus Erythematosus

**Figure 1:**
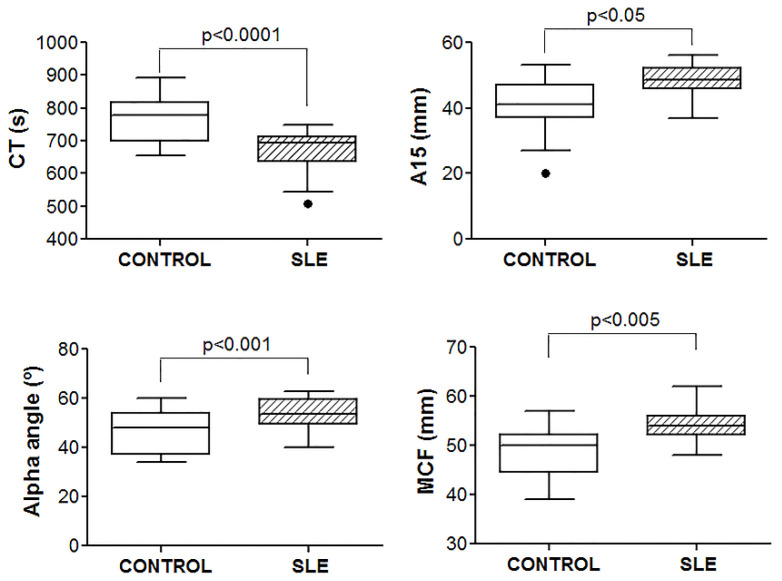
Procoagulant profile in patients with SLE. ROTEM^®^ thromboelastography was performed in whole blood. Detailed procedures and measured parameters are shown in “Materials and Methods”. Student’s *t*-test or Mann–Whitney test was performed, and *p* ≤ 0.05 was set as significant. SLE, systemic lupus erythematosus; CT, clotting time; A15, amplitude at 15 min; MCF, maximum clot firmness.

To evaluate the global hemostasis and kinetics of clot formation, a ROTEM^®^ test was performed using whole blood. Patients with SLE showed a procoagulant profile compared with the control samples (Figure 1). In the SLE patient group, we observed a shortening of the CT and a higher alpha angle, amplitude at 15 min, and MCF. No differences were found in the clot lysis at 60 min.

In order to determine whether MPs might participate in the procoagulant profile of patients with SLE, CAT was performed with different triggers that, according to the manufacturer, discriminate between thrombin generation dependent on the PS or TF content of MPs. As shown in Table 3, the ETP and peak of thrombin associated with the TF content of MPs was increased in patients with SLE. Moreover, the peak correlated to disease duration (Spearman r = 0.4634, *p* = 0.0084).

#### 3.1.3. Platelet Activation in Patients with Systemic Lupus Erythematosus

Platelets have an essential role in clot formation; thus, we tested whether they were involved in the prothrombotic profile observed in the thromboelastogram of patients with SLE.

**Figure 2:**
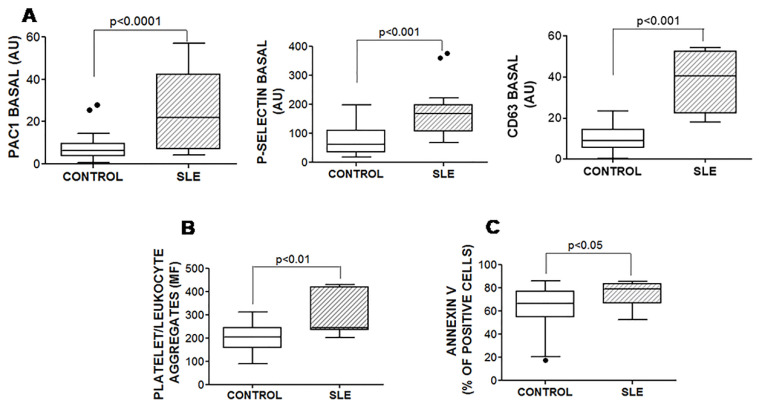
Basal activity of fibrinogen receptor, surface exposition of P-selectin, platelet/leukocyte aggregate formation and PS exposure. (**A**) PRP from healthy controls and patients with SLE was incubated with either FITC-PAC1, FITC anti-P-selectin mAb, or FITC anti-CD63 mAb. (**B**) To test the platelet/leukocyte aggregates, whole blood was incubated with PE anti-CD41 mAb and FITC-anti-CD45 mAb. (**C**) Annexin V binding was tested in washed platelets resuspended in the adequate buffer (see Methods). Samples were analyzed by flow cytometry. The Mann–Whitney test was performed, and *p* ≤ 0.05 was considered significant. Data are expressed as arbitrary units (mean fluorescence ×% of positive cells (**A**), mean fluorescence of leukocytes positive for CD41 (**B**), or percentage of positive cells (**C**)).

The platelets from patients with SLE presented basal activation, considering their increased PAC1 binding and major exposure of P-selectin and CD63 in quiescent conditions (Figure 2A). The basal activation of the platelets from patients with SLE was not the consequence of an increased expression of fibrinogen and vWF receptors on their surface (Figure S1). Moreover, we observed an increase in the platelet/leukocyte aggregate formation under basal conditions in the patients with SLE (Figure 2B). The percentage of platelet/leukocyte aggregate correlated with the ROTEM^®^ parameters MCF (Spearman r = 0.579, *p* = 0.030) and alpha angle (Spearman r = 0.532, *p* = 0.031) and with the basal P-selectin exposure on the platelet surface (Spearman r = 0.429, *p* = 0.035), but did not correlate with the platelet and leukocyte counts.The platelet activation in patients with SLE did not depend on the platelet count. Moreover, the ROTEM parameters did not correlate with platelet activation markers.

The response to activation with either 100 µM of TRAP or 20 µM of ADP was similar in the platelets from healthy controls and from patients with SLE (Figure S2).

#### 3.1.4. Phosphatidylserine Exposure and Apoptosis in SLE Platelets

Platelets from patients with SLE bound more annexin on their surface than the controls, indicating an enhanced PS exposure (Figure 2C). This fact did not appear to be related to enhanced apoptosis, because the caspase activities were similar among groups (Figure S3).

#### 3.1.5. Association between Coagulation Profile and Inflammatory State

**Figure 3:**
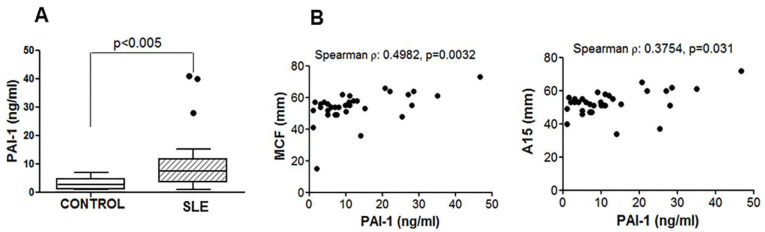
Plasminogen activator inhibitor-1 (PAI-1) levels in plasma and its correlation with ROTEM^®^ parameters. Plasma levels of PAI-1 (**A**) measured with enzyme-linked immunosorbent assay correlated with A15 and MCF parameters (**B**). A Mann–Whitney test and Spearman’s correlation were performed, and *p* < 0.05 was considered significant.

An association between coagulation and inflammatory states has been reported [10]. Therefore, we tested the plasma levels of PAI-1, which is considered a marker of vascular inflammation [11]. The PAI-1 levels were increased in patients with SLE (Figure 3A).

Furthermore, the PAI-1 levels correlated with the ROTEM^®^ parameters A15 and MCF (Figure 3B), suggesting an association between the PAI-1 plasma levels and the procoagulant state observed in patients with SLE. On the contrary, the PAI-1 levels did not correlate with the disease activity index.

#### 3.1.6. Thrombin Generation Associated with Neutrophil Extracellular Trap Formation

**Figure 4:**
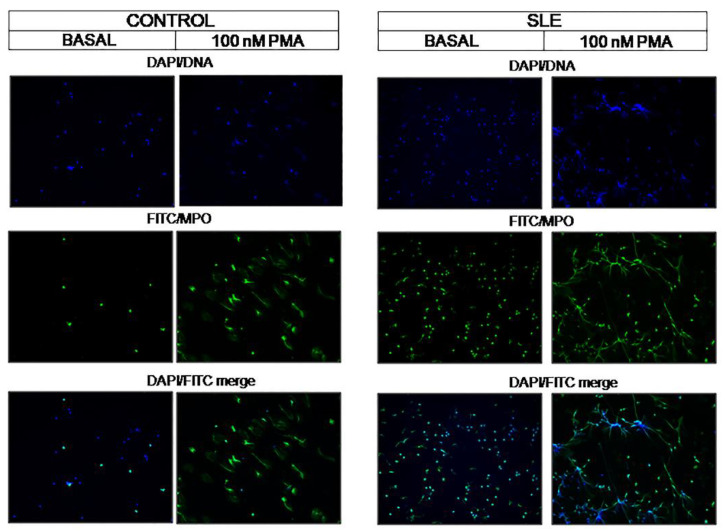
Formation of NETs.NETs were evaluated in basal conditions and after stimulation with 100 nMof PMA. DNA (DAPI, blue), neutrophil myeloperoxidase (MPO, FITC, green), and DAPI/FITC merge images areshown. Original magnification ×10.

The plasma content of nucleic acids might contribute to the creation of prothrombotic profiles. We observed that the plasma from patients with SLE had increased cfDNA in fluorescence units, controls: 94.90 ± 21.29, SLE patients: 112.4 ± 26.59; *p* = 0.0211). In accordance with this observation, the neutrophils from SLE patients, but not the controls, showed NETs in basal conditions (Figure 4). Moreover, the neutrophils from these patients generated more NETs in the presence of 100 nM of PMA, as confirmed by fluorescence microscopy (Figure 4).

**Figure 5:**
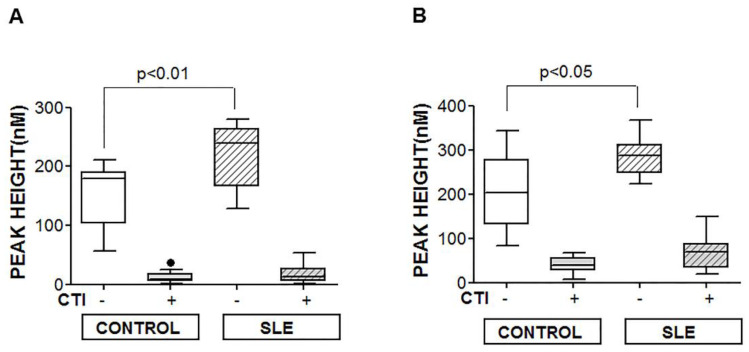
Thrombin generation associated with NETs. The effect of NETs on the thrombin generation was tested in either non-stimulated (**A**) or 100 nM of PMA-stimulated neutrophils (**B**) in the presence of PRP from healthy controls adjusted to 1 × 10^5^ platelets/µL, with (+) or without (−) CTI. Detailed procedures are explained in “Materials and Methods”. A Mann–Whitney test was performed, and *p* < 0.05 was considered significant.

To evaluate whether the increment in NETs observed in patients with SLE had consequences on the hemostasis of these patients, we tested the thrombin generation of neutrophils from either patients with SLE or controls in the presence of platelets from healthy controls. The neutrophils from patients with SLE produced more thrombin than those from healthy controls under basal conditions and after stimulation with 100 nM of PMA. These increments were avoided when PRP was collected from blood samples drawn with CTI (Figure 5).

## 4. Discussion

Analyses of the ROTEM^®^ parameters in our cohort of patients showed a shortened CT and an increased alpha angle and MCF, highlighting the hypercoagulable features of these patients despite the fact that they had no antiphospholipid antibodies and no history of suffering thrombotic events. However, K.S. Collins et al. [12], who tested the kinetics of clot formation employing another global method, thromboelastography (TEG), found no differences in the TEG parameters between patients with SLE and healthy controls. This discrepancy could be due to the trigger used to induce coagulation in each case. These authors employed kaolin to activate the clotting cascade. Kaolin is a more powerful trigger, but offers a minor sensitivity in detecting mild differences in coagulation kinetics. Differently, we used the non-activated rotational thromboelastometry, NATEM^®^, which is sensitive to any change in the balance of the coagulation system, but has a low specificity [13]. Interestingly, other authors have evaluated coagulation by TEG in a cohort of children with SLE, and they also found a procoagulant profile; in this study, however, patients with antiphospholipid antibodies were not excluded [14].

In some of the patients from our cohort, SLE was accompanied by other syndromes such as Raynaud and Sjögren, known to induce a procoagulant state [15,16] that might overlap with that produced by SLE. Nevertheless, this does not seem to occur, because no differences were found among groups of SLE patients without and with these accompanying syndromes.

We did not find a correlation between the activity index of the disease and the patients’ procoagulant profile. A similar conclusion was drawn from a systematic review and meta-analysis performed by Balloca et al. [17]. Despite the lack of correlation between the disease activity and procoagulant profile, we compared the clinical features of SLE patients with an MCF higher than the mean value + SD of MCF from healthy controls with those with an MCF within the normal range. We observed that, while both groups had similar nephrological damage, those with a high MCF had approximately 1.5 times more muscular and cardiac compromise and three times more pulmonary and nervous system clinical manifestations. Even when these data should be verified increasing the number of patients in the cohort, our observation warns about the importance of maintaining patients with hemostatic and coagulation parameters within normal ranges. Accordingly, some authors have proposed to treat patients with SLE with prophylactic oral anticoagulant therapy [18].

Platelet number and function are important determinants in the kinetics of clot formation [12,19]. The platelet counts in our SLE cohort, despite being significantly lower than in the healthy control group, were within a normal range. Thus, this difference was not expected to alter the ROTEM^®^ parameters and, if any effect was predictable, it was a hypocoagulable one.

Treatment in SLE aims at remission or low disease activity and the prevention of flares. Our cohort of patients with SLE was treated according to the EULAR recommendations [20]. In patients without treatment, disease was in remission. Nevertheless, due to the complex and diverse nature of SLE pathogenesis, most of the patients need a combinatory therapy that may include standard immunosuppressive drugs (corticosteroids, azathioprine, methotrexate, cyclophosphamide, and mycophenolate) and monoclonal antibodies blocking CD20 (rituximab) or B-cell-activating factors (belimumab). In particular, one of our patients received at the same time rituximab and belimumab. This combination has been recently tested and demonstrated to reduce immune-complex-mediated inflammation and NETs formation [21].

Most of the patients with SLE from our cohort were treated with hydroxychloroquine, a substance known to have inhibitory effects on platelets [22]. Nevertheless, we and other authors [23] have observed that platelets from patients with SLE are basally activated. This activation could be due, at least in part, to the effect of the anti-dsDNA antibodies present in patients with SLE that may induce platelet activation, demonstrated by the enhanced P-selectin expression and morphological platelet changes [24]. However, the platelets from patients with SLE showed a reduced exposure of P-selectin after TRAP and ADP stimulation. Similarly, Frelinger et al. had reported that platelets from children with immune thrombocytopenia showed an increased P-selectin exposure in quiescent conditions but not after stimulation with agonists [25]. This observation might be because the basal activation of platelets causes either a reduction in the number or the exhaustion of secretory α-granules.

Another consequence of the basal activation of platelets from patients with SLE is the enhancement of their interaction with leukocytes (present results and other authors [26,27]) through the binding of P-selectin with the PSGL-1 present on the leukocytes’ surface. Once bound, the interaction between the integrin αMβ2 on leukocytes and the GPIb on platelets produces a firm adhesion that contributes to thrombosis [28]. Moreover, platelet–leukocyte interactions induce signals that amplify proinflammatory cellular responses [29,30].

In accordance with their basal activation [31], platelets from patients with SLE exposed more PS than platelets from healthy controls (Figure 2C). Given that the PS exposition on the surface of platelets provides a negatively charged scaffold for the binding of the tenase and/or prothrombinase complexes that promote thrombin generation [32], it is tempting to speculate that this is an additional mechanism through which platelets could participate in the procoagulant profile of patients with SLE. The PS exposure on platelets from patients with SLE was due to activation and not to apoptosis [33], because the caspase activities in their quiescent platelets were similar to those observed in platelets from the healthy controls.

The procoagulant profile in patients with SLE might also be due to the presence of MPs released by cells in response to activation or apoptosis. Given that SLE is characterized by chronic inflammation and tissue damage, it is not surprising that blood from patients with SLE contains more MPs [34,35,36], due to both activated and damaged cells. MPs support coagulation by the exposure of negatively charged phospholipids and TF. We observed that our cohort of patients with SLE generated more thrombin associated with the TF content of MPs, whereas no differences were observed in thrombin generation linked to the PS content of MPs. These results might be explained because MPs from patients with SLE are predominantly PS-negative [37]. Nevertheless, Pereira et al. has reported an increased thrombin generation dependent on PS-associated MPs [36]. Moreover, other authors have described the augmentation of MPs in patients with SLE, measuring their PS exposure [38]. These differences might rely on the inclusion criteria for patients with SLE or/and the use of distinct technical approaches for measuring MPs. Given that the MPs in SLE exhibit unique molecular and phenotypic features, including the infrequent expression of PS, their role in the pathogenesis of SLE and their utility as biomarkers have been suggested by many authors [35,39].

On the basis of the existence of a correlation between the procoagulant profile (the peak of thrombin generation associated with MPs’ TFcontent) and disease duration (but not with the disease activity index), it is tempting to speculate that chronic damage is more important than the current disease activity for the hypercoagulable state of these patients. Nevertheless, it cannot be ruled out that lack of correlation between coagulation parameters and disease activity might be due to our small sample size.

The increase in TF-bearing MPs could come from damaged endothelial cells [40]. In support of this observation, we and other authors [35,39] have observed increased plasma levels of PAI-1, a marker of endothelial dysfunction, in patients with SLE. In addition, augmented PAI-1 accompanied higher MCF values.

The damage to endothelial cells in SLE could be due, in part, to the presence of elevated levels of a pathogenic neutrophil subset known as low-density granulocytes, which have been reported to contribute to lupus pathogenesis through heightened proinflammatory responses, altered phagocytic capacity, and vascular damage [41]. Moreover, this neutrophil subset tends to release more web-like NETs [42,43], which are composed of cfDNA, histones, antimicrobial proteins, fibrinogen, FXII, and TF [43,44]. This release explains the increased levels of cfDNA found in plasma from patients with SLE. Moreover, most of the patients with SLE have a reduced ability to degrade NETs [42], and the prolonged presence of NETs in plasma could promote a rupture of immune tolerance as well as increase tissue damage [45]. These events lead to the formation of an amplification loop, in which NET components induce autoantibodies, leading to the formation of more immune complexes which, in turn, perpetuate NET formation. The experiments of Etulain et al. performed on mice showed an increase in NETs in the presence of P-selectin and PSGL1 [46]. This could explain why the neutrophils from patients with SLE were more susceptible to generating NETs due to basal platelet activation.

Moreover, enhanced NET formation has been associated with the promotion of coronary plaque formation and lipoprotein dysregulation [47].

The increased thrombin generated by non-stimulated neutrophils from patients with SLE (present results) could be explained by the fact that cfDNA triggers the intrinsic pathway of blood coagulation [48]. NETs can bind FXII and cooperate with platelets to activate the intrinsic pathway [49]. In support of this observation, NET-related thrombin generation was prevented in the presence of CTI, an inhibitor of the contact phase of coagulation activation (present results and [50]).

One of the limitations of the study is that the patients were receiving different treatments that might modify hemostasis, as mentioned above for hydroxychloroquine, and that we did not recruit enough patients for stratifying them according to the medication they were receiving.

Traditional cardiovascular risk factors do not fully explain the high rates of ischemic events in patients with SLE, and standard risk calculations underestimate the risk of developing cardiovascular disease. Previous reports have already shown similar results to those presented in this work—platelets from SLE patients are activated [27] and increments were observed in circulating MPs [36], platelets/leukocyte aggregates [26], and PAI-1 [39] and cfDNA [51] plasma levels. Importance of our work relies on the fact that all these variables were evaluated in the same cohort of patients, allowing us to detect the relationship between the different mechanisms involved. In addition, the effects observed were independent of antiphospholipid antibodies because their presence was an exclusion criterion. Another key point is that our results suggest the utility of global tests for studying hemostasis in these patients, because a procoagulant profile was detected despite the fact that they had neither antiphospholipid antibodies nor any previous thrombotic event. A global appraisal of hemostasis takes into account the relationships among all the mechanisms involved (platelets, the thrombin generation associated with MPs, cfDNA) and should, if possible, be incorporated into clinical practice to detect the risk of a thrombotic event in patients with SLE and to consequently act to prevent its occurrence, as recommended in the updated guide of EULAR [20].

## Figures and Tables

**Table 1:** Features of the patients with Systemic lupus erythematosus (SLE).

Patient	Disease Duration (Years)	Age (Years)	Medication at the Time of the Study	Concomitant Diseases	SLEDAI-2K
1	23	44	No treatment		12
2	21	50	Omeprazole, mycophenolate mofetil, prednisone, calcifediol	Autoimmune thrombocytopenia	6
3	9	35	Tramadol, levothyroxine, prednisone, rituximab	Sjogren’s syndrome, Graves-Basedow disease, autoimmune hepatitis, fibromyalgia	3
4	18	35	Ramipril, phenelzine, abatacept, immunoglobulins		4
5	26	32	No treatment	Raynaud’s phenomenon, endometriosis,	1
6	19	31	No treatment	Photosensitivity, inflammatory arthralgias, lymphopenia	5
7	13	49	Ferrous sulfate, omeprazole, prednisone, belimumab, azathioprine	Sjogren’s syndrome	4
8	19	35	Quetiapine, duloxetine, omeprazole, diazepam, hydroxychloroquine, azathioprine, pregabalin, prednisone, tramadol, ferrous sulfate, calcifediol, rituximab		2
9	27	57	Prednisone, azathioprine, belimumab, rituximab		14
10	12	46	Calcifediol		2
11	4	45	Clobetasol propionate, trazodone, calcipotriol, diazepam, hydroxychloroquine, calcifediol, pregabalin, metamizole, omeprazole, almotriptan, enalapril, prednisone, sertraline, mycophenolate mofetil, abatacept, belimumab	Raynaud’s syndrome, lupus nephropathy, mixed dyslipidemia	2
12	10	29	Azathioprine, hydroxychloroquine, prednisone, omeprazole, calcifediol, ferrous sulfate, belimumab	Sjogren’s syndrome, leukopenia/lymphopenia	7
13	2	45	Hydroxychloroquine, prednisone, calcifediol, levothyroxine, azathioprine		0
14	15	33	Hydroxychloroquine, azathioprine, chondroitin sulfate		4
15	3	21	Hydroxychloroquine		4
16	23	61	Symbicort, enalapril, tramadol, pregabalin, azathioprine		0
17	10	31	No treatment		4
18	11	35	Methotrexate, omeprazole, folic acid, prednisone, mycophenolate mofetil		2
19	22	41	Nifedipine, hydroxychloroquine	Mixed connective tissue disease, Raynaud’s syndrome	2
20	24	56	Abatacept, furosemide, fluoxetine, lorazepam, amisulpride, omeprazole, prednisone, spironolactone	Rheumatoid arthritis, Sjogren’s syndrome, autoimmune hepatitis	4
21	6	67	Hydroxychloroquine, calcifediol		0
22	8	62	Hydroxychloroquine, omeprazole, levothyroxine, diazepam, paroxetine		2
23	23	58	Hydroxychloroquine, prednisone, atenolol, calcifediol		4
24	7	25	Hydroxychloroquine, calcifediol, Robaxisal		10
25	4	65	Hydroxychloroquine, prednisone, atorvastatin	Arterial hypertension	8
26	9	40	Mycophenolate mofetil, prednisone, hydroxychloroquine, enalapril, denosumab, ranitidine, paroxetine, calcifediol		0
27	8	22	Mycophenolate mofetil, ursodeoxycholic acid, hydroxychloroquine, calcifediol, ranitidine	Alpha-thalassemia minor, Raynaud’s syndrome, secondary hyperhidrosis	4
28	22	48	Hydroxychloroquine, prednisone, calcifediol, omeprazole, cholecalciferol		2
29	12	40	Calcifediol		2
30	18	33	Prednisone, hydroxychloroquine, calcifediol	Atrial septal aneurysm, Kikuchi-Fujimoto disease, osteonecrosis	2
31	37	52	Omeprazole, hydroxychloroquine, amitriptyline, calcifediol	Depression	4
32	15	38	Hydroxychloroquine		0

**Table 2:** Biochemical parameters in the healthy controls and patients with SLE.

	Controls	SLE	*p*-Value	Normal Range
Lymphocytes/µL	1.9 (1.6–2.4)	1.6 (1–1.8)	0.0093 *	1.2–3.4
Erythrocytes ×10^6^/µL	4.3 (4.1–4.6)	4.1 (3.8–4.4)	0.0421 *	4–6
Monocytes ×10^3^/µL	0.4 (0.3–0.5)	0.4 (0.2–0.4)	0.1046	0.1–0.6
Granulocytes ×10^3^/µL	4 (2.9–5.3)	2.9 (2.3–3.6)	0.0065 *	1.4–6.5
Leukocytes ×10^3^/µL	6.4 (5.3–7.6)	4.8 (4.2–5.7)	0.0012 *	4.5–10.5
Hemoglobin (g/dL)	13.2 (12.3–14.2)	12.9 (11.6–13.5)	0.1226	11–18
Platelets ×10^3^/µL	247 (208–284)	194 (171.5–231)	˂0.0001 *	150–450
Hematocrit (%)	40.1 (38.4–43.7)	35.9 (34.4–39.8)	0.0004 *	35–60
MCV (fL)	94.6 (91.5–96.9)	92.4 (88.1–97)	0.2407	80–99.9
MCH (pg)	30.3 ± 1.6	28.6 ± 3.3	0.3037	27–31
MCHC (g/dL)	31.1 (31.3–32.9)	31.6 (30.7–32.3)	0.0976	33–37
RDW (%)	13.5 (12.8 –14.3)	14.1 (13.2–15.5)	0.1561	11.6–13.7
MPV (fL)	6.9 ± 0.8	7 ± 0.8	0.8931	7.8–11
Pct (%)	0.17 (0.15–0.19)	0.14 (0.12–0.19)	0.1210	0.190–0.36
PDW (%)	17.1 ± 0.8	17.3 ± 0.9	0.4936	0.190–0.36
CRP (mg/dL)	n.d	0.2650 (0.12–0.6)	-	0–0.5
C3 (mg/dL)	n.d	88.4 (71.5–106)	-	75–135
C4 (mg/dL)	n.d	16.3 (11.8–20.7)	-	14–60
Anti-DNA (mg/dL)	n.d.	14 (2.9–23)	-	˂15.00
ESR (mm)	n.d	10.77 ± 0.6	-	2–20
Creatinine (mg/dL)	n.d	0.72 ± 0.12	-	0.5–0.9
IgG (mg/dL)	n.d.	1130 (910.5–1252)	-	725–1900
IgA (mg/dL)	n.d.	224.6 ± 92.32	-	50–350
IgM (mg/dL)	n.d.	84 (61.6–107.5)	-	45–280

Mann–Whitney or Student’s t-tests were performed, and data are expressed as median (percentile 25%–percentile 75%) or mean ± SD depending on the sample distribution. A *p*-value ≤ 0.05 was set as significant, and * denotes significance. MCV, mean corpuscular volume; MCH, mean corpuscular hemoglobin; MCHC, mean corpuscular hemoglobin concentration; RDW, red blood cell distribution width; MPV, mean platelet volume; Pct, plateletcrit; PDW, platelet distribution width; CRP, C-reactive protein; ESR, erythrocyte sedimentation rate; n.d., not determined.

**Table 3:** Microparticle-associated procoagulant capacity in patients with SLE.

	Controls	Patients with SLE	*p*
LT PRP-Reagent (min)	7.5 ± 3.8	7.8 ± 2.3	0.1280
Peak PRP-Reagent (nM)	90.5 ± 40.8	86.2 ± 52.5	0.1347
ttPeak PRP-Reagent (min)	12.1 ± 4.0	13.5 ± 3.2	0.1489
ETP PRP-Reagent (nM/min)	1021.0 ± 457.3	1107.0 ± 323.0	0.6076
LT MP-Reagent (min)	13.3 ± 3.2	13.2 ± 2.9	0.1186
Peak MP-Reagent (nM)	151.4 ± 36.5	253.8 ± 66.5	0.0021 *
ttPeak MP-Reagent (min)	16.1 ± 3.6	16.0 ± 3.3	0.1370
ETP MP-Reagent (nM/min)	1065.0 ± 241.8	1188.0 ± 313.6	0.0482 *

Data are expressed as mean ± SD. A *p*-value ≤ 0.05 was set as significant, and * denotes significance. Abbreviations: LT, lag time; ttPeak, time to peak; ETP, endogenous thrombin potential.

## Supplementary Materials

The following are available online at https://www.mdpi.com/2077-0383/9/10/3297/s1, Figure S1: Fibrinogen and von Willebrand factor (vW Factor) receptors expression on quiescent platelets. Platelets were incubated with PE-anti CD41 and FITC-anti CD61 mAbs to detect fibrinogen receptor and with FITC-anti CD42a and FITC-anti CD42b mAbs to test vW Factor receptors. Data are expressed as mean fluorescence (MF). Samples were analyzed by flow cytometry. Figure S2: Platelet’s activation markers. Platelets were stimulated with either 100 µM of TRAP or 20 µM of ADP, and FITC-PAC1, FITC-anti P-selectin mAb, and FITC-anti CD63 mAb were added. Data are expressed as % of positive cells. Samples were analyzed by flow cytometry. Figure S3: Caspase activities in quiescent platelets. Data are expressed as % of positive cells. Samples were analyzed by flow cytometry.
