# Supplementary material for: Insights into the Procoagulant Profile of Patients with Systemic Lupus Erythematosus without Antiphospholipid Antibodies
Source: J Clin Med. 2020 Oct 14;9(10):3297. doi: 10.3390/jcm9103297 (PMC7602183; doi:10.3390/jcm9103297)
Supplement: Supplementary file 1 [file jcm-09-03297-s001.pdf]

SUPPLEMENTARY MATERIAL

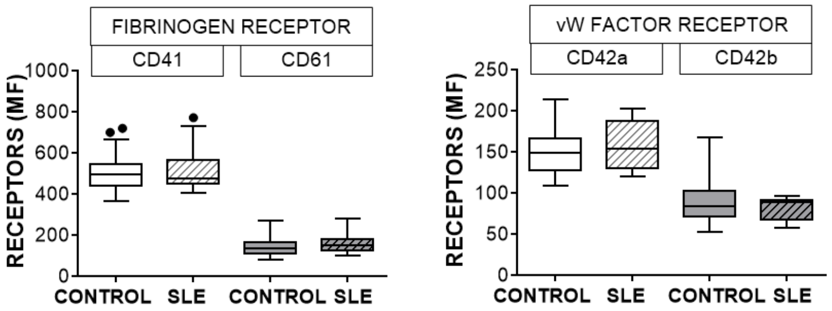

Figure S1. Fibrinogen and von Willebrand factor (vW Factor) receptors expression on quiescent platelets. Platelets were incubated with PE-anti CD41 and FITC-anti CD61 mAbs to detect fibrinogen receptor and with FITC-anti CD42a and FITC-anti CD42b mAbs to test vW Factor receptors. Data are expressed as mean fluorescence (MF). Samples were analysed by flow cytometry.

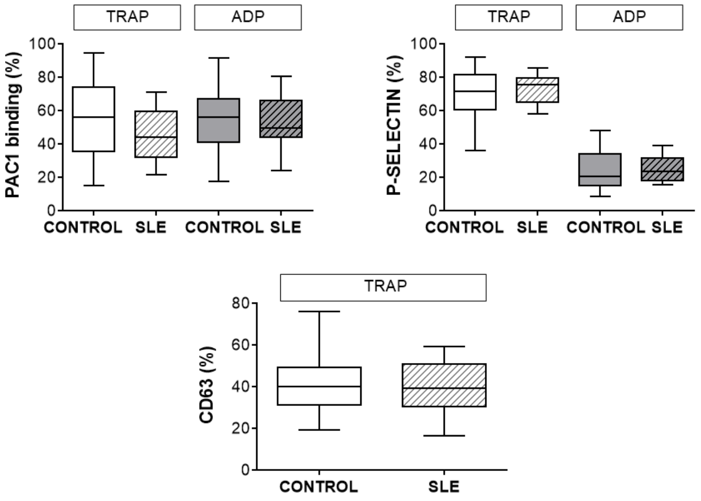

Figure S2. Platelet's activation markers. Platelets were stimulated with either 100 μM TRAP or 20 μM ADP and FITC-PAC1, FITC-anti P-selectin mAb and FITC-anti CD63 mAb were added. Data are expressed as % of positive cells. Samples were analysed by flow cytometry.

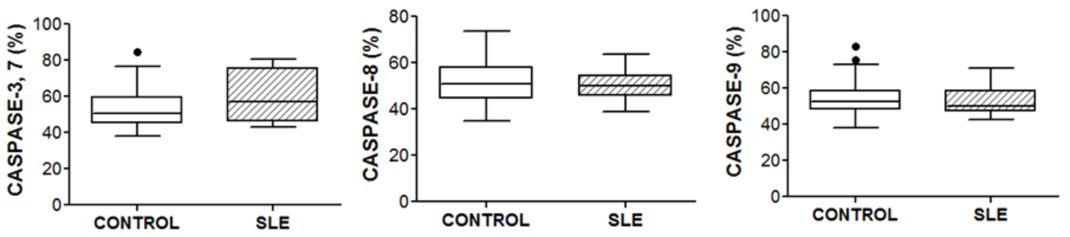

Figure S3. Caspase activities in quiescent platelets. Data are expressed as % of positive cells. Samples were analysed by flow cytometry.
